# Posterior Cruciate Ligament Tibial Avulsion treated with Open Reduction and Internal Fixation

**DOI:** 10.5704/MOJ.1507.008

**Published:** 2015-07

**Authors:** WXP Lee, MO Kyaw

**Affiliations:** Department of Orthopaedic Surgery, Sibu Hospital, Sibu, Malaysia

**Keywords:** Minimal invasive, Percutaneous pedicle screw fixation, thoracolumbar fractures, conservative treatment

## Abstract

The optimal treatment for thoracolumbar fractures (TLF) without neurological deficit remains controversial. Majority of the systematic reviews and meta-analyses have evaluated open operative approaches but have yet to compare the outcomes of minimally invasive percutaneous pedicle fixation (MIPPF) versus non-operative treatment. A retrospective cohort study was performed to compare clinical and radiological outcomes between MIPPF and conservative groups for TLF AO Type A1 to Type B2 during a 2-year follow-up period. Pre-operative plain and CT films were evaluated and decision made for short segment (non-fusion) MIPPF. Patients who refused operation were treated conservatively with three months of body cast, brace, or corset. MIPPF group showed earlier Visual Analog Score(VAS) improvement at six months post-injury (0 vs 6.0- p<0.001), as well as better functional and radiological outcomes (p<0.050) at final follow-up. Progressions of regional kyphosis (RK) were noted in both groups but there was no significant difference within and between them(p>0.050). MIPPF as a method of internal bracing can be pursued in the treatment of TLF, with larger future cohorts and RCTs being called for to support and explore new findings.

## Introduction

Thoracolumbar junction (T11 to L2) is the most common vertebral segment susceptible to fracture due to the transition of the relatively immobile rib-bearing thoracic vertebrae articulating with the more mobile lumbar vertebral segment. The optimal treatment for thoracolumbar fractures (TLF) without neurological deficit remains controversial. A vast number of TLF can be treated with traditional open approach of pedicle screw fixation. The advent of minimally invasive percutaneous pedicle screw fixation (MIPPF), originally aimed at treating degenerative spine pathologies, is increasingly being used in the last few decades to treat certain types of TLF^1-4^. MIPPF confers advantages of posterior musculature preservation with less blood loss, shorter operative time, lower infection risk, less postoperative pain, shorter rehabilitation time and shorter hospital stay compared to that of open approach^3,5,10,12^. Studies have demonstrated efficacy of minimal invasive percutaneous pedicle fixation as an option of management for AO Type A and Type B thoracolumbar fractures in neurologically intact patients^1-3,6^. Several systematic reviews and meta-analyses of non-operative versus operative treatment for TLF have been conducted^7,9^. Majority of the studies included in these reviews had evaluated all types of operative approaches, namely posterior, anterior, or combined antero-posterrior. The literature is however, lacking in the comparison of - MIPPF operative approach with that of conservative treatment. We therefore performed a retrospective cohort study to evaluate the clinical and radiological outcomes of operative treatment using MIPPF technique with that of non-operative treatment for TLF AO Type A1 to Type B2 in neurologically intact patients.

## Materials and Methods

Operative registry search for all MIPPF TLF cases performed from January 2009 March 2011 as well as patients' registry and database search for all TLF (T11 to L2) cases (AO Classification Type A1.3, A3.3, B2.3) treated conservatively during the same period were carried out. Pre-operative plain radiographic imaging of antero-posterior and lateral views and Computed Tomography (CT) films were evaluated - based on the AO Classification and decision made for short segment (non-fusion) percutaneous pedicle fixation (one level above and one level below the fractured vertebra). Patients who had refused operation were treated conservatively with bed rest, analgesia and immobilization. Methods of immobilization in the conservative group consisted of either body cast, corset or thoracolumbar orthosis for three months. Inclusion criteria were: traumatic spine fracture, thoracolumbar level fracture involving T11 to L2 levels, AO Spine Fracture Classification Type A1.3, A3.3 and B2.3, non-pathological fracture, age 18 and above, patient without neurological deficit. Exclusion criteria were: pathological fracture, fracture involving more than one vertebral level at thoracolumbar region, patients with neurological deficit. Two spinal systems had been used: Depuy Viper II and Medtronic Sextant II percutaneous cannulated screws. All the MIPPF surgery were performed by a single surgeon.

Patients' clinic follow-up records were examined for both clinical and radiological outcomes that were being monitored throughout regular outpatient follow-up. (1) pain, measured using Visual Analog Score (VAS)- (0 to 10, 0= no pain, 10= worst pain); (2) function and quality of life, measured using validated indices of Short-form (SF) 36 health survey (Physical Component Summary PCS Score, Mental Component Summary MCS Score). Radiological outcomes: (1) Cobb's angle of regional kyphosis measured in degrees (2) percentage of vertebral height compression in relation to that of adjacent one vertebral level cranial and caudal to the fracture level.

Data entry, cleaning and analyses were done using SPSS Version 18.0 for Windows. Descriptive statistics were used for baseline characteristics of both groups. Results for the outcomes of study were analyzed with non-parametric T-test Mann-Whitney test, Wilcoxon Signed Rank test, Spearman's Correlation test and reported with corresponding 95% confidence interval and p values. Less than 0.05 of p value was considered statistically significant. Clinical outcomes of VAS and SF 36 Physical and Mental Component Summary Scores were reported at six weeks, six months, 12 months and 24 months follow-up interval while radiological outcomes were evaluated at six weeks and at final follow-up. Approval for this study was obtained from the ethical committee of the institutional review board.

## Results

There was a total of 19 cases, 10 of which had undergone short segment MIPPF and nine patients had opted for conservative treatment. The average follow-up for MIPPF and conservative groups were 25 weeks and 28 weeks respectively. Table I shows that there were no significant differences in the baseline characteristics of the groups (p value >0.05).

**Table I:** Baseline Characteristics of the Patients

Characteristic	Total n (%) 19 (100.0)	MIPPF n (%) 10 (52.6)	Conservative n (%) 9 (47.4)	p value
Age (year) Median(IQR)	50 (22)	43.5 (25)	53 (23)	0.140
Gender-				
Male	17 (89.5)	8 (80.0)	9 (100.0)	0.167
Female	2 (10.5)	2 (20.0)	0 (0.0)	
Mechanism of Injury				
Fall from height	13 (68.4)	7 (70.0)	66.7	1.000
Motor Vehicle Accident (MVA)	3 (15.8)	1 (10.0)	22.2	
Others	3 (15.8)	2 (20.0)	11.1	
Fractured Vertebra Level				
T11	0	0	0	0.720
T12	5 (26.3)	2 (20.0)	3 (33.3)	
L1	10 (52.6)	6 (60.0)	4 (44.4)	
L2	4 (21.1)	2 (20.0)	2 (22.2)	
AO Classification (of fractured Vertebra)				
A 1.3	4 (21.1)	2 (20.0)	2 (22.2)	0.591
A 3.3	5 (26.3)	2 (20.0)	3 (33.3)	
B 2.3	10 (52.6)	6 (60.0)	4 (44.4)	

The MIPPF group demonstrated better clinical outcome- (p <0.050) compared to the conservative group throughout the 2-year follow-up period - (Table II). Additionally,, the former also showed better radiological outcome in terms of regional kyphosis (RK) and percentage of vertebral height (VH) compression at six weeks post-injury and at last follow-up (p <0.050) (Table III). Interestingly, the degrees of RK in both groups increased at last follow-up from the initial baseline six weeks post-injury period. However, there was no significant difference in the progression of RK within each group (MIPPF- p= 0.261; conservative- p= 0.812) and between the two groups (p= 0.389)). There was a rise in the percentage of VH compression in the MIPPF group at the modalities last follow-up but its progression did not show significant difference (p= 0.074) (Table IV).

**Table II:** Clinical Outcomes of MIPPF versus Conservative Groups for TLF

Clinical Outcome Variable		MIPPF n = 10 Median (IQR)	Conservative n = 9 Median (IQR)	Z statistic	p value
At 6 weeks	VAS*	4.5 (2)	7 (3)	-2.36	0.018
	PCS**	74.38 (34.44)	23.75 (10.63)	-3.51	<0.001
	MCS***	90.38 (10)	43.75 (27.85)	-3.18	0.001
At 6 months	VAS	0.0 (2)	6.0 (4)	-3.29	0.001
	PCS	96.50 (5.78)	58.75 (23.75)	-3.68	<0.001
	MCS	96.10 (3.31)	71.88 (20.17)	-3.52	<0.001
At 12 months	VAS	0.0 (0)	2.0 (3)	-3.13	0.002
	PCS	97.50 (3.9)	72.50 (10.94)	-3.69	<0.001
	MCS	96.73 (3.31)	88.25 (7.69)	-3.69	<0.001
At 24 months	VAS	0.0 (0)	2.0 (1)	-3.20	0.001
	PCS	97.50 (3.9)	72.50 (30.94)	-3.57	<0.001
	MCS	96.73 (3.31)	88.25 (7.81)	-3.16	0.002

* Visual Analog Score

** Physical Component Summary Score

*** Mental Component Summary Score

**Table III:** Radiological Outcomes of MIPPF versus Conservative Groups for TLF

Radiological Outcome Variable		MIPPF n = 10 Median (IQR)	Conservative n = 9 Median (IQR)	Z statistic	p value
At 6 weeks	RK¶	5.50 (11.00)	19.00 (15.00)	-2.94	0.003
	VH⊙	14.55 (16.42)	52.38 (24.17)	-3.35	0.001
At 24 months	RK	8.00 (8.00)	23.00 (16.00)	-2.45	0.014
	VH	24.53 (12.91)	45.45 (19.46)	-3.11	0.002

¶ Regional Kyphosis (Cobb’s angle in degree)

⊙ Vertebral Height (Percentage of compression)

**Table IV:** Progression of Regional Kyphosis and Percentage of Vertebral Height Compression within each Group and Between MIPPF Conservative Groups

Variable	Groups	Within Each Group		Between Two	Groups
		Z Statistics	p value	Z Statistics	p value
RK Progression Median (IQR)	MIPPF 3.50 (10.50)	-1.12	0.261	-0.86	0.389
	Conservative 1.00 (8.00)	-0.24	0.812		
VH Progression Median (IQR)	MIPPF 4.39 (13.05)	-1.78	0.074	-1.63	0.102
	Conservative 1.05 (13.48)	-0.30	0.767		

We were interested to assess if there was any relationship between several variables within each groups at the last follow-up. Based on Spearman's Correlation Test, there was no relationship between PCS Score with the degree of regional kyphosis in both groups (Table V). In the conservative group, there was a positive correlation between age and percentage of VH compression r^2^ = 0.239. However the relationship was not statistically significant (p = 0.535). There was a negative correlation between MCS Score and degree of regional kyphosis, r^2^ = -0.208 in the MIS group but there was no statistical significant difference, (p = 0.564) (Table V).

**Table V:** Variable Correlations within MIPPF & Conservative Groups

Variable Correlation at 24 months	r^2^	Groups MIPPF p value	Conservative r^2^	p value
VH⊙ with Age	-0.298	0.403	0.239	0.535
PCS** with RK¶	0.209	0.562	0.259	0.500
MCS*** with RK¶	-0.208	0.564	0.450	0.224

⊙ Vertebral Height (Percentage of compression)

** Physical Component Summary Score

*** Mental Component Summary Score

¶ Regional Kyphosis (Cobb’s angle in degree)

**Fig. 1a:**
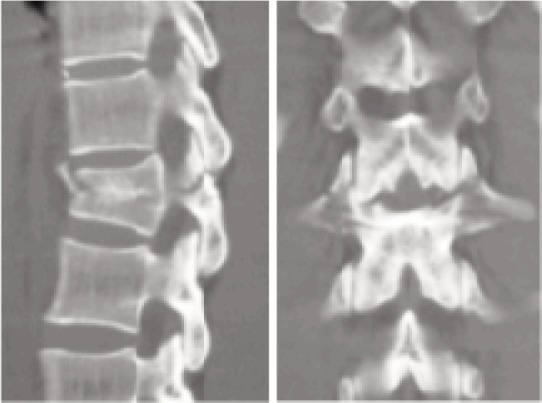
Pre-operative CT sagittal and coronal views of L1 fracture (AO Type B 2.3).

**Fig. 1b:**
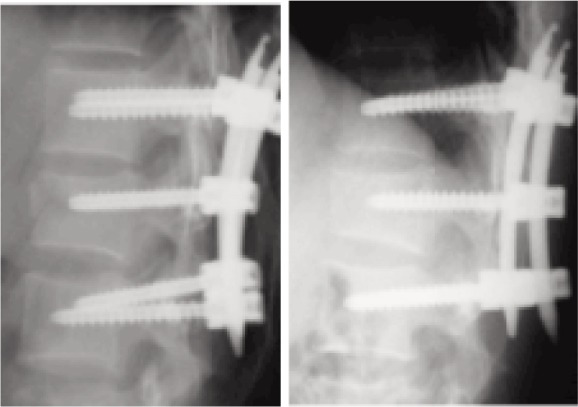
Post MIPPF radiological view-rediographs at 6 weeks and at 2 years.

**Fig. 2:**
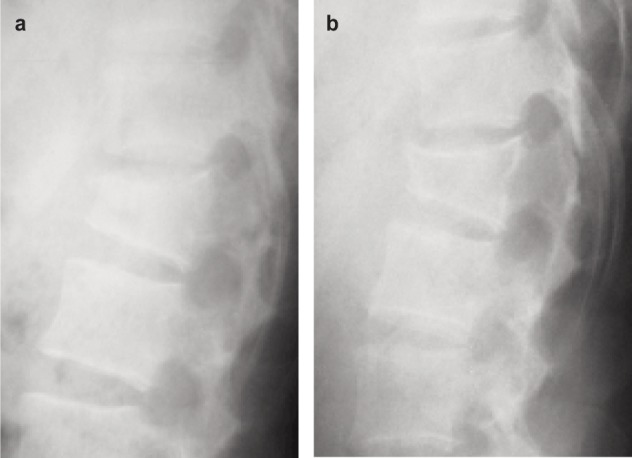
Lateral films at 6 weeks (2 (a)) and 6 months (2 (b)) post-injury of a case of L1 fracture (AO Type B 2.3) treated conservatively.

## Discussion

Systematic reviews and meta-analysis on the treatment of TLF with and/or without neurological injury have not shown sufficient clear evidence demonstrating its optimal treatment modalities^9,13-16^, hence the lack of consensus in its decision-making. We are aware that in recent years, MIPPF is increasingly being used in the treatment of TLF as a method of internal bracing. However, evidence in the literatures on the efficacies of this method of treatment for spine trauma is limited to a small case series and case reports^3,17-21^. Small comparison studies between open surgical techniques and percutaneous pedicle screw placement have demonstrated that the latter has the same advantages as the former which include restoration of sagittal alignment and stabilization of fractures but with less of the morbidities associated with open exposures such as high intra-operative blood loss, prolonged duration of surgery, increased infection rates and paraspinal muscle denervation or injury^3-5,10-12^. As for the non-operative group, proponents advocating this option pointed out the morbidities and complications associated with open stabilization approaches, hence the unnecessary subjection of patients to operation in view of absence of significant differences in the outcome being observed in the long term^7,9^. While some patients may benefit from conservative approach, the requirement for prolonged bedrest, compliance issues with cast or brace may not be feasible for the majority of patients. Moreover, there are potential complications of progressive spinal deformity, persistence of pain and occasional neurological compromise^1-3^. Therefore, in our study, we were interested in evaluating whether MIPPF would be a good option in combating the challenges of conservative management in the treatment of neurologically intact TLF without subjecting patients to the potential morbidities of traditional open approaches.

Our study showed statistically significant difference in the VAS scoring of MIPPF group at baseline until last follow-up with earlier improvement being observed as well at six months post-injury (0 vs 6.0- p < 0.001). Yi *et al*^9^ in their Cochrane Systematic review concluded that there was no statistically significant difference in the pain and function related outcome two years or more post-injury between operative and non-operative treatments for TLF without neurological deficit. Sonali *et al*^7^ in their meta-analysis on non-operative versus operative treatment for TLF without neurological deficit also demonstrated no differences in VAS pain score and functional outcome between the two groups at last follow-up. The functional outcome of SF- 36 PCS Scores and MCS Scores in our study showed significant differences (p < 0.050) in the MIPPF group throughout the follow-up period. At the last follow-up, the MIPPF group demonstrated better functional outcome compared to those in the conservative group (PCS: 97.50 (3.9) vs 72.50 (30.94)- p <0.001; MCS: 96.73 (3.31) vs 88.25 (7.81)- p =0.002). The contrasting results of our study compared to those mentioned in the reviews may be attributed to the difference in methods of surgical approaches in the operative groups, i.e. MIPPF versus traditional open approaches. Their meta-analysis^7^included all operative groups regardless of the types of surgical treatment, namely traditional open posterior, anterior, or combined approaches with or without fusion. To our - knowledge, there is no comparative study in the literature between the conservative group and the relatively recent MIPPF used specifically as a method of internal immobilization in the treatment of TLF, which thereby poses to us as limitation to allow for comparison of our current results.

Although there were significant differences in radiological outcome (RK and VH) between MIPPF and conservative group with the former demonstrating better outcome both at baseline and at last follow-up (MIS: 8.00 (8.00) vs 23.00 (16.00) (p = 0.014), progression of kyphosis was noted to occur in both groups. Reid *et al*^22^ reported that the kyphotic progression appears to occur in the initial post-injury period, with relative stabilization of kyphosis noted within 12 to 18 months. In our study, we did not evaluate the radiological parameters at 12 months post-injury. Chaichana *et al*^23^ in their literature review reported that the MIPPF technique is of advantage to expeditiously immobilize a thoracolumbar fracture with minimal or no kyphosis. Our further statistical analysis however did not show significant differences in the progression of RK within and between the two groups. Moreover, our study showed that there was no significant association between degree of kyphosis and functional outcomes in both groups. This is consistent with findings from other studies^7,24-31^. The VH and RK would be expected to progress more in the group of osteoporotic patients. Association test between age and vertebral height compression showed a positive correlation in the conservative group but was not of statistical difference when there was no relationship demonstrated in the MIPPF group. These findings have implications in justifying the clinical importance of kyphosis as a common outcome measure in other studies^28-34^.

In this study, there was no complication documented in the MIPPF group. No cases of infection, loss of fixation, fracture non-union and neurological compromise were reported. In the conservative group, compliance rate to body cast and brace posed - limitations. We are unable to comment on whether there was any significant difference in the clinical and radiological outcomes using different methods of external bracing and casting. We acknowledge the limitations of our study in being a retrospective study with small sample size. We could not perform subgroup analysis based on fracture types due to small sample size. Points to note in the MIPPF group are the issues of cumulative exposures to radiation to both surgeons and patients alike, as well as the costs of cannulated percutaneous screw systems. The drawback of radiation exposure in fluoroscopic-assisted MIPPF is undoubtedly a concern not to be taken lightly. Navigation system or computer-assisted surgery system available today may be advantages in reducing irradiation time and exposure^35^. Technical issues in MIPPF surgery call for a learning curve with the prerequisite of ample surgical experience in traditional open posterior approach where direct visualization of anatomical landmarks may assist in accuracy of screw placement and reduce the likelihood of pedicle wall violation thereby shortening operating time and complications of general anaesthesia. Patient selection is paramount in that the risks and benefits should be weighed out particularly for those with underlying co-morbidities that may potentially preclude them from undergoing surgery under general anaesthesia while bearing in mind the possible complications if treated conservatively. The cost of MIPPF implants remains a point of debate. Within the proper context, one can perhaps rationalize that earlier functional improvement and the return to work in the MIPPF group could be overall cost-effective. However, future cost-analysis studies in this regard may be worthwhile.

## Conclusion

Our study showed that minimally invasive percutaneous pedicle screw fixation as a method of internal bracing can be pursued in the treatment of thoracolumbar fractures. Comparison between minimally invasive operative group and conservative group demonstrated better functional and radiological outcomes with statistically significant differences in the MIPPF operative group at baseline and throughout the follow-up period. In the light of this, future larger comparative cohorts and randomized controlled studies are called for to support our study results and perhaps explore new findings that will be of greater benefit in contributing to the decision-making for treatment of thoracolumbar fractures.
